# Electroporation safety factor of 300 nanosecond and 10 millisecond defibrillation in Langendorff-perfused rabbit hearts

**DOI:** 10.1371/journal.pone.0257287

**Published:** 2021-09-24

**Authors:** Johanna U. Neuber, Andrei G. Pakhomov, Christian W. Zemlin

**Affiliations:** 1 Department of Electrical and Computer Engineering, Old Dominion University, Norfolk, VA, United States of America; 2 Frank Reidy Research Center for Bioelectrics, Old Dominion University, Norfolk, VA, United States of America; 3 Division of Cardiothoracic Surgery, Washington University School of Medicine, St. Louis, MO, United States of America; University of Minnesota, UNITED STATES

## Abstract

**Aims:**

Recently, a new defibrillation modality using nanosecond pulses was shown to be effective at much lower energies than conventional 10 millisecond monophasic shocks in ex vivo experiments. Here we compare the safety factors of 300 nanosecond and 10 millisecond shocks to assess the safety of nanosecond defibrillation.

**Methods and results:**

The safety factor, i.e. the ratio of median effective doses (ED50) for electroporative damage and defibrillation, was assessed for nanosecond and conventional (millisecond) defibrillation shocks in Langendorff-perfused New Zealand white rabbit hearts. In order to allow for multiple shock applications in a single heart, a pair of needle electrodes was used to apply shocks of varying voltage. Propidium iodide (PI) staining at the surface of the heart showed that nanosecond shocks had a slightly lower safety factor (6.50) than millisecond shocks (8.69), p = 0.02; while PI staining cross-sections in the electrode plane showed no significant difference (5.38 for 300 ns shocks and 6.29 for 10 ms shocks, p = 0.22).

**Conclusions:**

In Langendorff-perfused rabbit hearts, nanosecond defibrillation has a similar safety factor as millisecond defibrillation, between 5 and 9, suggesting that nanosecond defibrillation can be performed safely.

## 1. Introduction

The delivery of intense electric shocks has for decades been the principal life-saving intervention to terminate ventricular fibrillation. Adverse effects of defibrillation may include increased morbidity and mortality, anxiety, pain, and cell damage [1–3]. These effects increase with the amount of energy deposited during treatment. The search for more efficient yet safer defibrillation has brought the transition from monophasic to biphasic waveforms [4–6] and motivates the ongoing research into low-energy defibrillation strategies [7–9].

One approach to lowering defibrillation energies is to shorten the shock duration. Advances in pulsed power engineering as well as increased understanding of the bioeffects of ultrashort electrical pulses has led to the investigation of nanosecond pulsed electric fields for various biomedical applications, including tumor ablation [10–14] and, more recently, cardiac ablation [15]. Neural and cardiac stimulation by nanosecond pulses also have been demonstrated [16, 17]. A natural concern is whether such short, intense shocks lead to tissue death via electroporation or other electrophysiological effects [18]. Recently, we have shown that 300 ns pulsed electric fields are capable of defibrillation at energies of about an order of magnitude lower than 10 ms monophasic shocks [9]. In the same study, we also showed that single defibrillation-strength shocks caused neither PI uptake nor tissue death (as determined with a tetrazolium chloride stain) and that the only immediate electrophysiological effect was a prolongation of the diastolic interval directly following the shock.

Further studies in cardiomyocytes measured PI uptake at 5 times the stimulation threshold for various pulse durations, with 200 ns shocks resulting in 1.7 times less uptake than 800 ns shocks and 4 times less than 10 μs shocks [17]. To determine whether shorter pulses also create less damage in myocardial tissue, we investigated how the field strength required for electroporation (*E_EP_*) compares to the field strength required for defibrillation (*E_Defib_*); the ratio of these two field strengths is called the “safety factor”, *SF* = *E_EP_/E_Defib_*. The field *E_EP_* is defined as ED50 of electroporation, i.e. the field that causes electroporation in 50% of the cells, as indicated by PI uptake. Similarly, *E_Defib_* is defined as the field that accomplishes defibrillation in 50% of the attempts, i.e. ED50 of defibrillation.

A complication in the design of this study was that while electric shock delivery with parallel plate electrodes may be the best approximation of clinical defibrillation, this geometry exposes the whole heart to the applied electric field and allows for only one shock application per heart, resulting in an excessive use of rabbits. To avoid this limitation, we inserted a pair of needle electrodes into the myocardium in order to apply fields in a localized manner. As a result, we were able to place about 10 applications per heart. To find the relationship between the shock-induced fields in the needle electrode and parallel plate electrode geometries, we used electrostatic modeling of both geometries.

## 2. Methods

### 2.1 Surgical preparation

The IACUC of Old Dominion University (Protocol Number: 15–015) approved the animal protocols for the experiments reported here. All animal experiments were performed in accordance with the NIH guidelines [19]. New Zealand white rabbits of either sex (3–4 kg, n = 9) were heparinized (500 IU/kg) and brought to a surgical plane of anesthesia with 4–5% isoflurane by inhalation. The heart was rapidly removed, the aorta cannulated and flushed with ice cold Tyrode’s solution (in mM: NaCl: 128.2, NaCO_3_: 20, NaH_2_PO_4_: 1.2, MgCl_2_: 1.1, KCl: 4.7, CaCl_2_: 1.3, glucose: 11.1). The heart was then placed in a Langendorff-perfusion setup, where it was perfused and superfused with warm oxygenated Tyrode’s solution (37±0.5°C, 7.4±0.05 pH) at a constant pressure of 60–80 mmHg.

### 2.2 Electrode configuration

**Fig 1:**
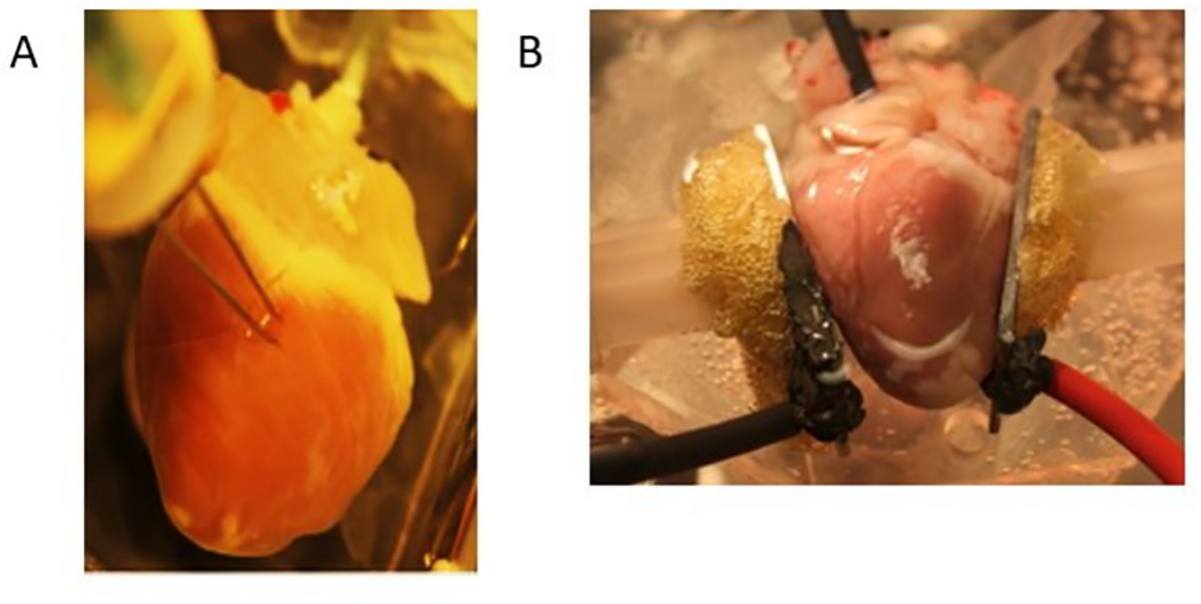
Electrode configurations for defibrillation and safety factor determination. A) View of the needle electrodes penetrating into the myocardium. B) View of the parallel plate electrodes for determining the defibrillation threshold.

Two parallel tungsten needles (Ø 250 μm) were inserted into the epicardium. The electrode spacing was 4 mm, and the electrodes were insulated with a hemisphere of silicone with the terminal 4 mm exposed (see Fig 1A). We took care not to damage any of the main coronary vessels (right coronary artery, left anterior descendant artery, left circumflex artery). The fine needles were only left in place until all shocks were applied (~ 1 min), and the minor leaks created should not negatively affect the tissue in a Langendorff preparation. To mark the insertion points, the electrode tips were dipped into surgical ink before being inserted into the epicardium. Electrode insertion points were at least 1 cm apart, and placed on both the posterior and anterior walls of both ventricles, allowing for up to 18 sequential applications per heart. The previous electrode configuration (Fig 1B) used parallel plate electrodes and only allowed for one application per heart.

### 2.3 Computational modeling of electric field distribution

**Fig 2:**
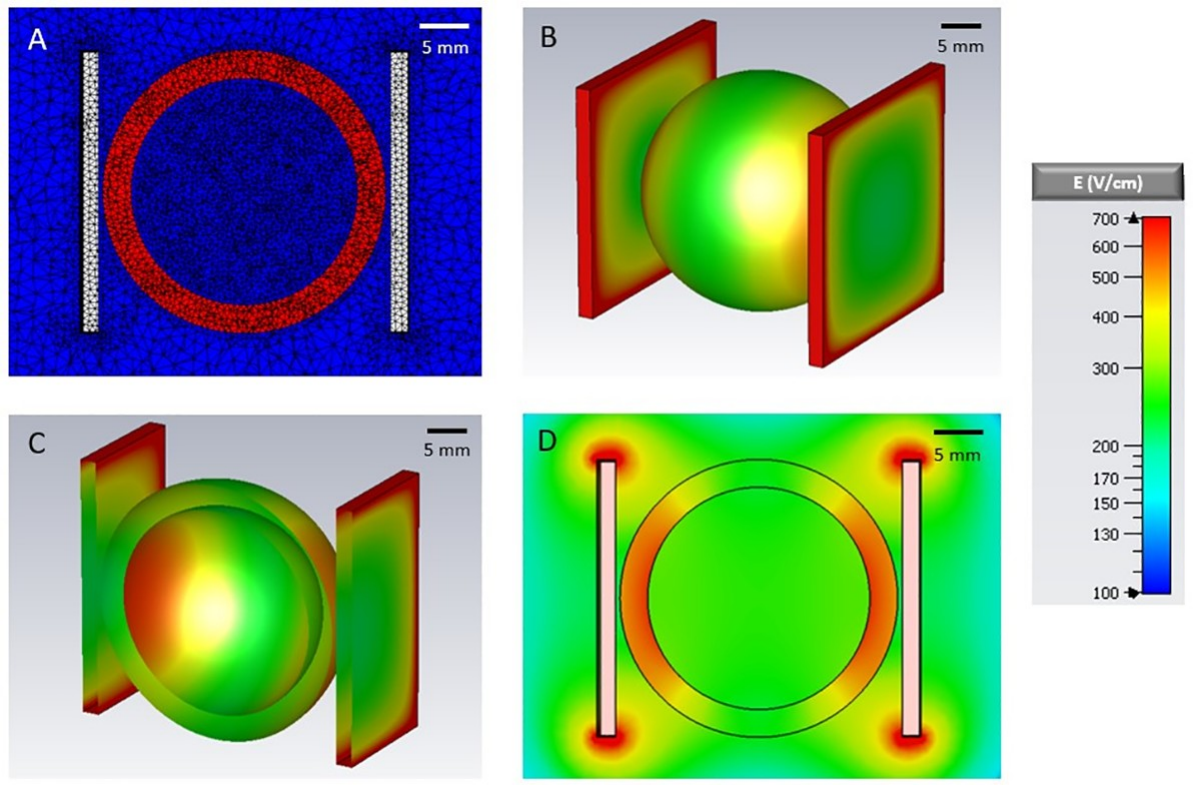
Electric field distribution for a voltage of 1 kV applied to two parallel plate electrodes (separation 2.8 cm) positioned on either side of a simplified heart model. A) 2D mesh view of the xz-plane B) 3D view of the closed cardiac shell model C) The model cut open along the xz-plane and D) 2D view of the xz-plane.

To compute the static electric field distribution in the parallel plate electrode geometry that we used in prior defibrillation experiments, a spherical shell (12 mm inner radius, 15 mm outer radius) of cardiac tissue was modeled in between two perfect conductors of dimensions 30 mm x 30 mm x 1 mm as shown in Fig 2. One plate was set to +V/2 and the other to -V/2, where V is the desired potential difference between the two. The electrodes and the heart were immersed in a solution with the same electrical properties as the Tyrode’s solution used in our experiments. As shown in Fig 2D, the highest field strength was observed at the inner surface of the cardiac shell closest to the electrodes.

**Fig 3:**
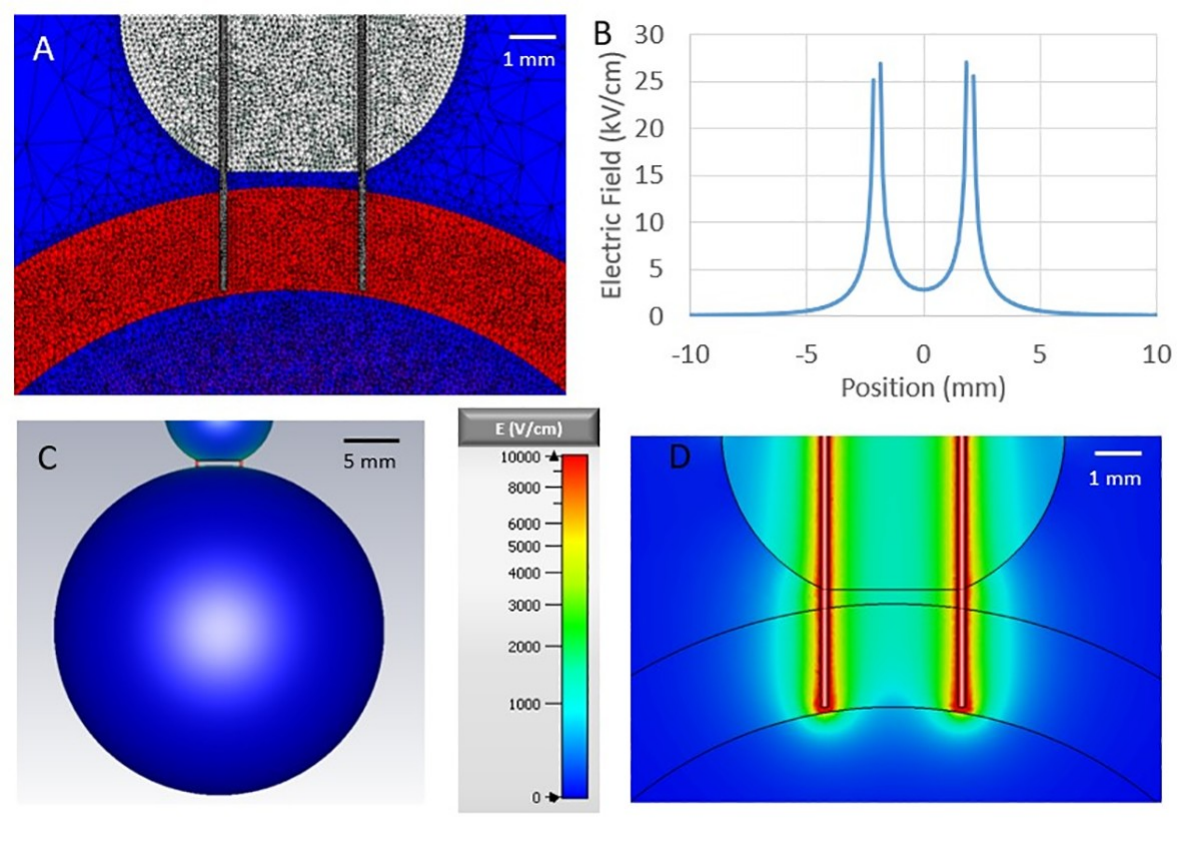
Electric field distribution for 1 kV applied to two needle electrodes inserted into the cardiac tissue of our simplified heart model. A) 2D mesh view of the cross section through the needle electrodes B) field strength along the line perpendicular to the long axes of the electrodes, at a tissue depth of 1.5 mm halfway between the electrodes C) 3D view of the cardiac surface and D) cross-section through needle electrodes.

In the penetrating electrode configuration (Fig 3), the electrodes were modeled using two rods of 0.12 mm radius with a 4 mm separation. The rods penetrated the spherical shell to a depth of 3 mm and were modeled as perfect electrical conductors. A dielectric hemisphere of 5 mm radius was also placed surrounding the sections of needle not inserted into the heart to model the silicone used to insulate and keep the needles in place. One needle was set to +V/2 and the other to -V/2, where V was the desired potential difference between the two.

We used the Computer Simulation Technology (CST) Microwave Studio® 3D Electromagnetic simulation software, specifically the electrostatic solver with tetrahedral meshing, to model our system. The solver uses the finite element integration to discretize Laplace’s equation, ∇∙σ∇*V* = 0, where V is the electric potential and σ is the conductivity. We neglected any permittivity effects, since the dielectric relaxation time constant is much shorter than our pulse duration. The relaxation time is given by $\tau_{D}=\frac{\varepsilon_{0}\varepsilon_{r}}{\sigma }$, where ε_0_ is the vacuum permittivity and ε_R_ is the relative permittivity of the material, leading to *τ_D_*≈ 15 ns [20], compared to a pulse duration of 300 ns. While conductivity generally depends on frequency, we approximated the conductivity of Tyrode’s solution as σ_T_ = 1.59 S/m [21–23] and the conductivity of cardiac tissue as σ_C_ = 0.6 S/m [20]. Note that the solution of the Laplace equation depends on only the ratio σ_T_/σ_C_, so variations in σ_T_ or σ_C_ with frequency do not affect our solution as long as they have the same frequency dependency. We also note that while the heart is anisotropic, here we only use the transversal conductivity of cardiac tissue. The axial conductivity is about an order of magnitude higher than the transversal conductivity. The rationale for using the transversal conductivity is that cardiac fibers, for all their complex architecture, run approximately parallel to the cardiac surface, and for the middle part of Fig 2D, this means that the electric field “sees” the transversal conductivity. At the top and the bottom of our spherical heart, the cardiac fibers could be oriented so that the electric field sees either the transversal conductivity (if the fibers are oriented into the paper in Fig 2D) or axial conductivity (if the fibers are oriented left-to-right in Fig 2D). This suggests we should also consider higher conductivities in the top and bottom of our heart model. Higher conductivity will lead to lower electric fields, and since these regions are already the experiencing the lowest electric field, a further reduction will have no influence on the predicted areas of electroporation, which are of course at the highest electric fields.

Fig 2A shows the tetrahedral mesh of the parallel plate configuration. The mesh is adaptive; in areas with larger variability in the material properties it is finer, and in areas with lower variability, it is coarser. The edge length varies from 0.02 to 4 mm, and the total number of tetrahedrons exceeds 1,000,000 in all cases.

The electric field distributions for the parallel plate configuration are shown in Fig 2B–2D. The maximum field strength (E_max,DV_) for the ED50 defibrillation voltage (DV) for the parallel plate configuration occurs on the inner surface of the cardiac sphere closest to the electrodes (the enhancement of the electric field at the edges of the electrodes does not extend to the cardiac shell). The relationship between the applied voltage and E_max,DV_ is linear, and the proportionality factor between the applied voltage and the corresponding electric field turns out to be 0.63 /cm.

Fig 3 shows the field distribution in our needle electrode model. Panel A shows the mesh, which now includes the needle electrodes and an electrode holder close to the cardiac surface (see also panel B). Fig 2C and 2D show the field distribution in the plane defined by the needle electrodes. Note that the field strength increases sharply towards the electrodes, so that for any applied voltage, the different regions of the tissue are exposed to a broad range of electric field strengths. We generally express the strength of electric fields in units of the E_max,DV_, the maximum field that any part of the heart experiences when a 1xDV shock is applied in the parallel electrode configuration (see above). Since electric field distributions in both geometries are governed by the linear Laplace equation, the field strength at any point in the heart is linear with respect to the applied voltage: For example, if a 2xDV shock is applied in the parallel electrode configuration, the maximum field strength occurring anywhere in the heart is 2 E_max,DV_.

To achieve the best estimation of the electroporation ED50, we applied voltages of amplitudes that resulted in different degrees of staining. Since the electric field drops off with distance from the electrodes, higher applied voltages result in a larger radius of staining, and the effect of different voltages can be combined to determine ED50 with greater accuracy. Initial modeling and experimental results showed that applying the ED50 defibrillation voltage for the parallel plate electrodes (DV) to the needle electrodes resulted in a staining radius of slightly less than 1mm (for both 10 ms and 300 ns shocks). Since the needle electrodes were separated by 4 mm, the stained regions around the two electrodes were clearly separated in this scenario. An applied voltage of twice the parallel plate ED50 defibrillation voltage (2xDV) resulted in a staining radius of approximately 1 mm. For an applied voltage of 5xDV, the staining radius typically exceeded 2 mm, and the stained regions around the electrodes began to overlap. These degrees of staining for 1xDV, 2xDV, and 5xDV were conducive to an accurate determination of ED50, so these shock amplitudes were used in the staining experiments. The defibrillation thresholds determined previously for the parallel plate geometry were 37 V for 10 ms shocks and 2.35 kV for 300 ns shocks across an electrode spacing of 3 cm [9]. The voltages applied to the needle electrodes were 37 V, 74 V, and 185 V for 10 ms and 2.35 kV, 4.7 kV, and 11.75 kV for 300 ns. All experiments had a 47 Ω resistor placed in series for impedance matching between the load and the pulsers. There was some variability in applied voltages (typically ~5–10%) due to the limited accuracy in the control of the charging voltage. To account for this variability, we experimentally measured the applied voltage for each shock and used the results to scale our model calculations.

### 2.4 Nanosecond and millisecond shock generation

Nanosecond shocks were delivered using the Pulse TX generator from Pulse Biosciences (Hayward, CA). The generator was capable of supplying shocks of amplitudes from 2 to 15 kV with durations varying from 200 ns to 1 μs.

Millisecond shocks were generated using a custom-made pulse generator consisting of a capacitor bank charged by a DC power supply (Kepco Power Supply Model APH 1000M). The pulse was shaped by a digital delay/pulse generator (Directed Energy PDG-2510).

**Fig 4:**
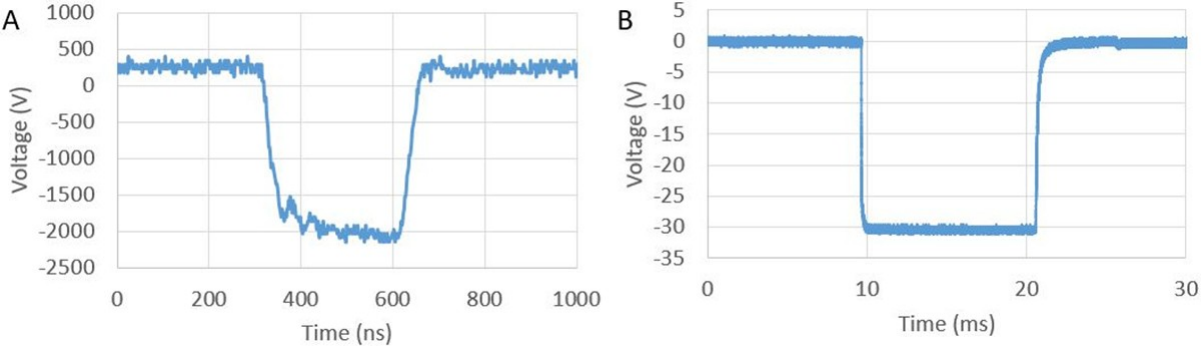
Shock waveforms. A) Representative waveform for 300 ns shock. B) Representative waveform for 10 ms shock.

Voltage waveforms were captured for both nanosecond and millisecond shocks using the internal PulseTX oscilloscope and a Teledyne Lecroy Wavesurfer 10M oscilloscope, respectively. Representative waveforms can be seen in Fig 4A (300 ns shock) and Fig 4B (10 ms shock).

### 2.5 Shock application and PI imaging

Treated hearts were perfused with PI (10 mg/mL, Sigma Aldrich) diluted in Tyrode’s solution (30 μM) for the entirety of the experiment. PI can enter cells with permeabilized membranes (but not those with intact membranes). After entering the cell, the dye binds to DNA or RNA and becomes strongly fluorescent.

After five minutes of equilibration to allow the heart to stabilize in the setup, the electrode needles were inserted perpendicular to the myocardial surface at 10 to 16 locations and a single shock was applied at each location. In total, the treatment duration was between 10 and 15 minutes For the 9 experiments performed, the 10 ms and 300 ns treatments were performed in alternating starting order, with randomized application of the three shock strengths within each set of shock durations. Additionally, one to two sham exposures were performed in each heart, where the electrodes were inserted into the myocardium but no shock was applied. After treatment, PI perfusion continued for 30 minutes to ensure that post-shock perfusion for both the first and last lesions was similar and any post-shock cell death was captured. The hearts were then flushed with cold (0°C) Tyrode’s solution to arrest the heart and flush out any remaining PI. The hearts were then placed in formalin for at least 30 minutes to fix the tissue.

An Olympus SZX16 microscope with a FITC cube was used for quantifying PI fluorescence, and images were captured using a Hamamatsu C9100 EM-CCD camera in conjunction with the HCImageLive software, version 4.4.0.11. The surface of the heart was imaged at each shock application site. Additionally, each individual lesion was excised using a scalpel with a minimum border of 1 mm undamaged tissue in all directions, and a longitudinal cross section of the lesion, intersecting both electrode points of insertion and perpendicular to the myocardial surface, was imaged. In some cases, when the tissue was not adequately sectioned along the midplane of the electrodes, a further, transverse cut was performed at each electrode insertion point, and the resulting surface was then imaged. Pixel sizes were 15–18 μm/pixel.

### 2.6 Electroporation ED50 calculation

From the electric field distribution that was calculated for the needle electrode geometry, electric field values were extracted on the plane that intersects the electrode axes and the surface of the cardiac shell in proximity to the electrodes. These values were then used to determine the electric field values at each pixel location of the experimental fluorescence images. To illustrate the shape of the electric field, we also extracted several isolines (i.e. lines of a constant value of the electric field).

To quantify the electroporation ED50 electric field for the surface images, we used the needle electrode field distribution and bilinearly interpolated to calculate the electric field at each pixel location (using ImageJ). Pixels with at least twice the background fluorescence level were considered above damage threshold. We then determined the fraction of pixels above the damage threshold as a function of electric field strengths, using increments of 5 V/cm for the 10 ms shocks and 120 V/cm for the ns shocks. The increments were chosen such that the threshold can be determined with a small number of measurements (5–10), using our experience from preliminary experiments. The electroporation ED50 was then taken to be the electric field value where 50% of the pixels were stained. This method mimics a commonly used way to define the defibrillation ED50 [24]. It should be noted that some of the staining is caused by reversibly electroporated cells, but given the steep drop of the electrical field of a needle electrode as the distance is increased, only a narrow ring of tissue should experience reversible electroporation, and this tissue is going to be damaged in varying degrees.

For the cross-sectional images, the cutting plane often differed slightly from the plane of electrode insertion. Thus, the fluorescence intensity along the electrode axis was evaluated to determine the tissue depth at which the fluorescence intensity was the strongest. At this depth, the fluorescence signal was quantified along a line (7 pixels or 0.14 mm wide) perpendicular to the electrode. ED50 calculations are performed as above, with binning increments increased to 10 V/cm for the 10 ms shocks and 200 V/cm for the ns shocks due to the smaller number of pixels.

### 2.7 Statistical analysis

Once the safety factor was determined for each cross-section or surface staining image, the results for 300 ns and 10 ms were separately averaged and the standard deviation and standard error of the mean (SEM) were calculated. Group results are presented as mean ± SEM. These results were compared using two-tailed, unpaired Student’s t-tests where p<0.05 was considered statistically significant.

## 3. Results

### 3.1 Surface PI staining

**Fig 5:**
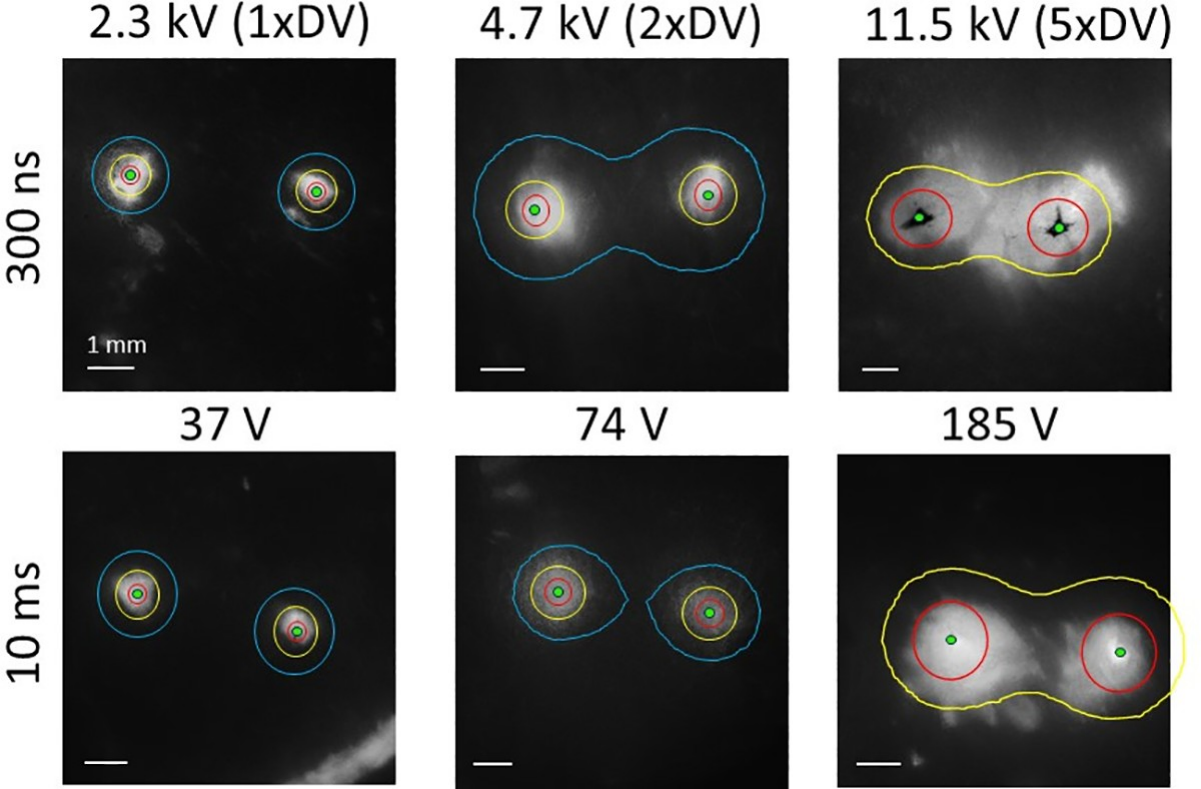
PI staining of cardiac surface for 300 ns and 10 ms shocks of different field strength. A) Representative PI staining examples for both 300 ns and 10 ms with applied voltages corresponding to 1, 2, and 5 defibrillation thresholds at 1 mm from the electrodes. The isolines show where the field strength equals to 15 E_max,DV_ (red), 6 E_max,DV_ (yellow), and 3 E_max,DV_ (blue). In the case of the strongest applied voltage, the 3 E_max,DV_ isoline is omitted because it runs mostly outside the field of view. B) Safety factor for the surface staining results for 300 ns (n = 26) and 10 ms shocks (n = 23). The averages are denoted with an “x”, the box shows the first quartile to the third quartile with the median intersecting the box, and the whiskers denote the maximum and minimum.

As shown in Fig 5A, there was an increase in stained area as the applied voltage increased for both microsecond and nanosecond shocks. The isolines depicted showed 15 E_max,DV_, 6 E_max,DV_, and 3 E_max,DV_ from inside to outside. It can be seen that for all durations, the 6 E_max,DV_ isoline approximately corresponded to the area of staining. However, there was some anisotropy observed, particularly at the highest voltage application for each shock duration. This held true for most of the analyzed images and was likely caused by the anisotropic structure of cardiac tissue.

Evaluation of 26 applications of 300 ns shocks and 23 applications of 10 ms shocks resulted in an average safety factor of 6.50±0.51 for 300 ns shocks and 8.69±0.80 for 10 ms shocks. This difference was statistically significant (p = 0.02).

### 3.2 Cross-sectional PI staining

**Fig 6:**
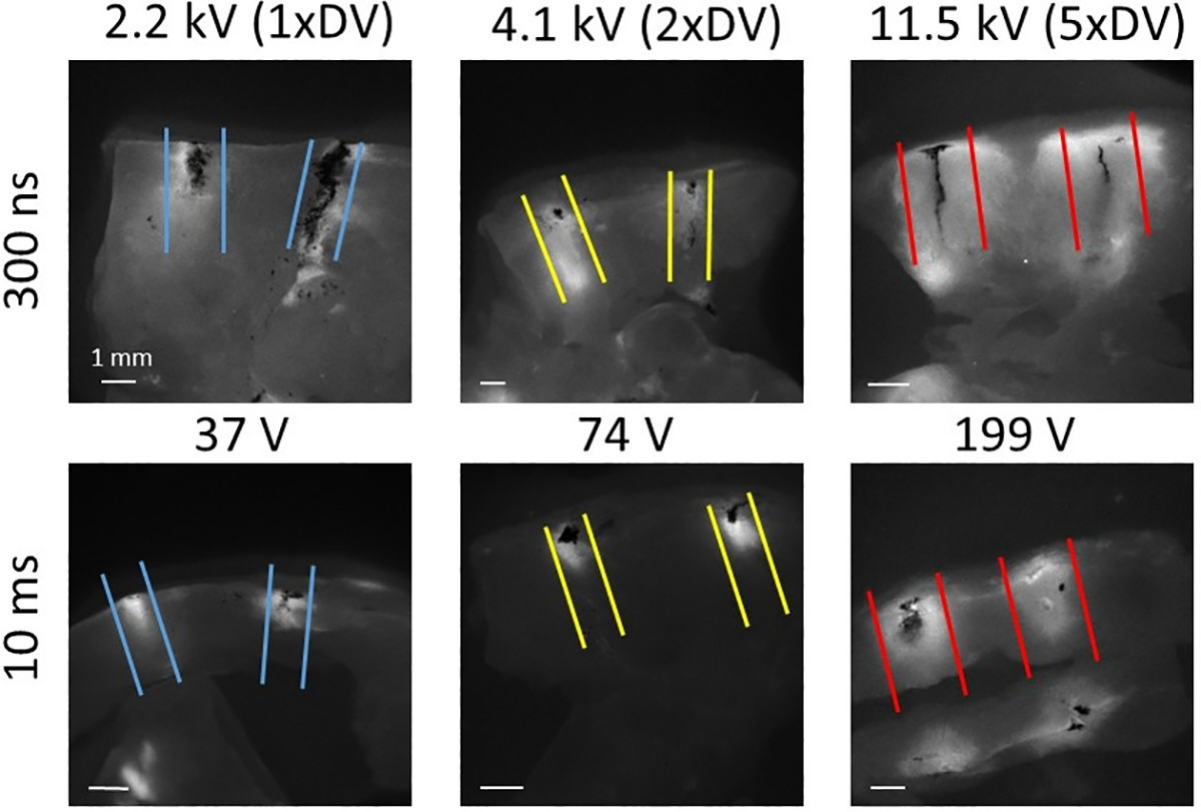
PI staining of tissue cross sections for shocks of different field strength. A) Representative examples for PI staining in the needle plane, for both 300 ns (top) and 10 ms (bottom). The parallel lines denote the isolines for 3 (blue), 6 (yellow), and 15 (red) E_max,DV_. B) Safety factor for the cross sections for 300 ns (n = 18) and 10 ms shocks (n = 17). The averages are denoted with an “x”, the box shows the first quartile to the third quartile with the median intersecting the box, and the whiskers denote the maximum and minimum.

Additionally, in 7 out of 9 experiments, cross-sections of the tissue in the electrode plane were imaged (see Fig 6). Quantitative analysis was performed as for surface staining. In this case, the safety factor was 5.38±0.52 for 300 ns shocks and 6.29±0.51 for 10 ms shocks, and the difference was not significant (p = 0.22).

## 4. Discussion

We have shown that the safety factors of 300 ns pulses and 10 ms defibrillation are comparable. In isolated rabbit hearts, shocks were delivered with a pair of penetrating electrodes, and there was a slight difference in electroporation as determined by propidium uptake. The numerical values of the safety factors as determined by surface staining were about 6.5 for nanosecond and about 8.7 for millisecond defibrillation. Their determination relied on computer simulations that related the electric field distributions for parallel plate electrode stimulation and penetrating electrode stimulation.

### 4.1 Surface versus and cross-section analysis

Our analysis of surface staining yielded a safety factor of 6.50 for 300 ns shocks and 8.69 for 10 ms shocks (p = 0.02). The analysis of cross sections gave a safety factor of 5.38 for 300 ns shocks and 6.29 for 10 ms shocks (p = 0.22). Both analyses have advantages and drawbacks. For surface analysis, fluorescence at the site of interest was easily detected, but the surface was slightly deformed by the insertion of electrodes, which distorts the electric field. Deformation was not a concern for the cross-section analysis, but it was harder to ensure that fluorescence was measured precisely in the electrode plane. Overall, we observed greater consistency in the cross-section analysis and have greater confidence in those results. Combining the results of both analyses, we conclude that the safety factor is in the range from 5 to 9 for both nanosecond and millisecond shocks with a trend to a higher safety factor for millisecond shocks that was not consistently statistically significant.

### 4.2 Implications for nanosecond defibrillation

Experiments were conducted with a penetrating electrode configuration to allow for multiple shock applications per heart, and computer modeling was used to select shock amplitudes from a parallel electrode configuration that is more representative of how electric fields are applied during defibrillation. The proper interpretation of these results requires some discussion of the modeling approach. In simulations of the parallel plate geometry, we observed the strongest field at those parts of the endocardium that face the electrodes (see Fig 2). It needs to be kept in mind that the propidium-stained areas that were determined in Fig 5 did not directly quantify the damage to be expected with defibrillation in the parallel-plate configuration. While the fact that significant propidium uptake started at fields around 6 E_max,DV_ indicates that we should expect to see damage at 6xDV for parallel plate electrodes, the extent of the expected damage also needs to consider the field distribution in the parallel plate configuration as shown in Fig 2. If, for example, 7xDV was applied, Fig 2 shows that in the walls facing the electrodes, the inner parts, up to about the center of the wall, was exposed to more than 6 E_max,DV_. We would therefore expect that this portion of the walls may be damaged and would take up propidium if we performed an experiment in this electrode configuration. The cardiac geometry used in Fig 1 is an approximation, but a similar interpretation is necessary in more detailed reconstructions of cardiac anatomy. Note that these considerations do not affect our conclusion that the safety factors of millisecond and nanosecond defibrillation are similar, which was based purely on the penetrating electrode data.

Similar safety factors of nanosecond and millisecond defibrillation may actually translate into nanosecond defibrillation being preferable if field uniformity is also considered. Several studies have suggested that the fields induced by nanosecond shock are more uniform that those of millisecond shocks [25, 26]. The field strength of a defibrillation shock needs to be chosen such that it excites the bulk of the tissue. For a more uniform field this should mean that less tissue is exposed to fields exceeding the damage threshold.

### 4.3 Comparison to earlier results on electroporation with millisecond pulses

While the results reported here are the first comparison of defibrillation and electroporation of cardiac tissue with nanosecond pulses, electroporation after millisecond shocks has previously been studied.

Wang et al. showed that for an internal coil-shaped electrode with a reference electrode not touching the heart, PI staining after defibrillation was only observed close to the internal active electrode [27]. In this report, the highest electric field was next to the active electrode and decreased with distance. This is a different geometry than our needle electrode configuration, but is consistent with our findings that electroporation was only seen close to the needle electrodes where electric field is higher than at greater distances.

Additionally, Al-Khadra et al. determined the defibrillation and electroporation thresholds for a coil electrode placed in the ventricular cavity with the second electrode 2 cm away [1]. The shock waveform was a monophasic 8 ms truncated exponential. They found a safety factor of about 1.3, substantially below what we report here, but this should be expected since the coil electrodes used by Al-Khadra generated a much more heterogeneous electric field. Since defibrillation requires a certain minimum field almost everywhere in the heart, including in areas where the heterogeneous field is low, the areas where the heterogeneous field is high will experience a much higher field and therefore be more likely to exhibit electroporation.

The similar safety factors of nanosecond and millisecond shocks are unexpected, however, in light of recent experiments that exposed single cells to shocks of 200 ns to 2 ms duration [17]. In these cells, it has been shown that for shocks at 5 times the stimulation threshold, cells take up the least amount of PI for a 200 ns shock duration when compared to 800 ns, 50 μs, and 2 ms. However, even for 200 ns shocks, there was still significant PI uptake, and repeat excitation of cardiomyocytes at the stimulation threshold using nanosecond shocks is not possible without electroporative damage. It should be noted that in whole hearts, the defibrillation threshold is almost 6 times higher than the stimulation threshold for 300 ns [9, 28]. Thus, for whole heart experiments, there must be other factors at play that cause the safety factors of 300 ns and 10 ms to be similar and much greater than one.

### 4.4 Scaling considerations and practicality of nanosecond defibrillation

The results presented here for electroporation with shocks delivered via needle electrodes in rabbit hearts should be similar to what identical shocks would yield in human hearts. The structure of cardiac tissue is similar across mammals, and this structure, together with the time-dependent applied field, determines the current flow during applied shock and ultimately the field across cell membranes, which can then cause electroporation. Similarly, defibrillation relies on the activation of cardiomyocytes via shock-induced transmembrane fields, and the activation mechanism is also very similar across mammals [29].

Field strengths required for defibrillation are similar across mammalian species, which means that applied voltages need to be scaled linearly with the size of the heart or the thorax. Defibrillation success depends not only on spatial distribution of the applied field, but also on the electrophysiological state at the moment of shock application; this explains that the same shock strength in the same heart can sometimes defibrillate and sometimes fail to defibrillate.

Before nanosecond defibrillation can be considered in a clinical setting, additional research is necessary, both for external and internal defibrillation. For external defibrillation, with electrodes placed on the thorax, it needs to be determined how the shocks affect other exposed tissues, particularly the lung. With respect to internal defibrillation, it would be a considerable engineering challenge to build a small, battery-driven nanosecond pulse generator.

### 4.5 Limitations

In our data analysis, we pooled the data from different hearts, assuming that the random subject factor would not be significant. While this common practice in ablation research [30–32] and we balanced the number of nanosecond and millisecond ablations in each heart, a multifactor model to assess the significance of the random subject factor would be the definitive way to justify our assumption. We were not able to perform this multifactor analysis because the metadata associating the fluorescence data with the heart identities were inadvertently deleted in the data analysis process.

## 5. Conclusion

We tested the safety factors of nanosecond defibrillation in comparison to millisecond defibrillation in isolated rabbit hearts using needle electrodes and found little difference. Surface staining results show a safety factor of 6.50 for 300 nanosecond shocks and 8.69 for 10 ms shocks, p = 0.02. Cross-sectional results show a safety factor of 5.38 for 300 ns shocks and 6.29 for 10 ms shocks, p = 0.22. When combined with our previous results that nanosecond shocks can defibrillate at lower energies, nanosecond defibrillation appears to be a viable option for low-energy defibrillation.
